# Nonadherence to immunosuppressives and treatment in kidney transplant: ADHERE BRAZIL Study

**DOI:** 10.11606/s1518-8787.2021055002894

**Published:** 2021-05-20

**Authors:** Elisa Oliveira Marsicano-Souza, Fernando Colugnati, Sabina De Geest, Helady Sanders-Pinheiro

**Affiliations:** I Universidade Federal de Juiz de Fora Hospital Universitário Unidade de Transplante Renal Juiz de ForaMG Brasil Universidade Federal de Juiz de Fora. Hospital Universitário. Unidade de Transplante Renal. Juiz de Fora, MG, Brasil; II Núcleo Interdisciplinar de Estudos e Pesquisas em Nefrologia Juiz de ForaMG Brasil Núcleo Interdisciplinar de Estudos e Pesquisas em Nefrologia (NIEPEN). Juiz de Fora, MG, Brasil; III University of Basel Institute of Nursing Science Department of Public Health Basel Switzerland University of Basel. Institute of Nursing Science. Department of Public Health. Basel, Switzerland; IV KU Leuven Academic Centre for Nursing and Midwifery Department of Public Health and Primary Care Leuven Belgium KU Leuven. Academic Centre for Nursing and Midwifery. Department of Public Health and Primary Care. Leuven, Belgium

**Keywords:** Patient Nonadherence, Medication Nonadherence, Kidney Transplant, Multicenter Study, Health Services Accessibility

## Abstract

**OBJECTIVE:**

To estimate the prevalence and variability of nonadherence to immunosuppressives and nonpharmacological treatment across kidney transplantation centers and two health access-disparate regions in Brazil.

**METHODS:**

In a cross-sectional design, a random multistage sample of 1,105 patients was included, based on center transplantation activity (low/moderate/high) and region (R1: North/Northeast/Mid-West; and R2: South/Southeast). Nonadherence to immunosuppressives (implementation phase) was assessed using the Basel Assessment of Adherence to Immunosuppressive Medications Scale (BAASIS)^©^. Self-report questionnaires assessed nonadherence to physical activity, smoking cessation, alcohol intake, and appointment keeping. We compared regions using the adjusted-χ^2^ or t-test.

**RESULTS:**

Most patients were men (58.5%), white (51.4%), and had a mean age of 47.5 (SD = 12.6) years. Regarding kidney transplantation centers, 75.9% were from R2 and 38.2% had low activity. The patients in R2 were older, white-majority, had more frequently steady partners, and received peritoneal dialysis. Nonadherence to immunosuppressives ranged from 11–65.2%; 44.5–90% to physical activity; 0–23.7% to appointment keeping; and 0–14% to smoking cessation. The total prevalence of nonadherence and by region (R1 *versus* R2) were: for immunosuppressives, 39.7% (44.9% *versus* 38.1%, p = 0.18); for smoking, 3.9% (1% *versus* 5%, p < 0.001); for physical activity, 69.1% (71% *versus* 69%, p = 0.48); for appointment keeping, 13% (12.7% *versus* 12%, p = 0.77); and for alcohol consumption, 0%.

**CONCLUSION:**

Despite differences among centers and high variability, only the nonadherence to smoking cessation was higher in the region with greater access to kidney transplantation. We suppose that differences in healthcare access may have been overcome by other positive aspects of the post kidney transplantation treatment.

## INTRODUCTION

After kidney transplantation (KT), it is essential that transplant patients, who are considered chronically ill, adequately follow the proposed treatment to reduce the risks of graft rejection and the progression of existing comorbidities, and the development of new ones. KT treatment is complex and involves adherence to immunosuppressives and nonpharmacological treatment components (regular physical activity, smoking cessation, reducing alcohol intake, and appointment keeping)^1-3^.

The prevalence of nonadherence to immunosuppressives is 28–67% depending on casefinding methods, operational definitions, and measurement tools^1,4^. A seven-fold increase in graft failure, as well as a clear association with acute rejection episodes, worse graft function, higher morbidity, and higher costs to health systems, have been reported^5,6^. Fewer studies have assessed the adherence to nonpharmacological treatment in KT. The reported prevalence of nonadherence to physical activity is 21.8–84%, which is also driven by variability in methods^7^. Inadequate physical activity can increase the risk for cardiovascular disease, which consequently relates to mortality and lower graft survival^9,10^. For smoking, the prevalence is 2.8–4.0 cases per 100 patients per year^7^. This behavior is also associated with cardiovascular diseases, and smoking cessation is recommended upon the diagnosis of chronic kidney disease^10^. Concerning alcohol consumption, post-transplant abuse is associated with poor medication adherence, which may increase the risk of graft loss and death^11^. Another fundamental but rarely studied behavior is the frequency of appointment keeping. Ranging from 2.5 to 14.6%, nonadherent patients had a 1.5-fold increased risk of acute rejection and a 65% higher chance of graft loss^7,12^.

The ecological model proposes that adherence to these complex behaviors is a result of the interaction of multilevel factors, placing the patient at the center and influenced by factors from the healthcare provider/family (micro-), the transplant center (meso-), and the health care system (macro-) levels^12^. Some studies have corroborated this framework in transplantation^13,14^. While most reports have focused on patient-level factors, variables related to the micro- and meso-levels have been understudied. Should these factors be modifiable, they could be potential targets for interventions. For example, multidisciplinary follow-up for KT recipients based on the chronic care model, with continuous assessment of adherence as the “5^th^ vital sign” may be cited, as well as the provision of support for self-management by health care professionals such as nurses, pharmacists, or physical therapists, to decrease nonadherence to immunosuppressives and physical inactivity^15,16^.

Brazil is a country of continental extension that ranks second in the absolute number of KTs worldwide, performed by the largest public transplant program^17^. However, even with a full-coverage healthcare system, there are regional disparities regarding access to KT. Examination of the Brazilian Transplant Registry^18^ revealed two distinct regions based on transplant activity: the South/Southeast states with elevated organ donation and transplantation; and the Northeast, Mid-West, and North states showing lower performances^18^. Out of the 5,923 KTs performed in Brazil in 2018, 3,111 were performed in the Southeast, 1,457 in the South, 1,032 in the Northeast, 245 in the Mid-West, and 78 in the North^18^. Data regarding access to general health services shows the same profile. Access to healthcare services has been easier in the South and Southeast states, with a higher number of medical appointments and doctors. Greater access is also associated with the higher grades of socioeconomic parameters statuses of these regions^19,20^.

The numbers of KT centers and healthcare professionals’ teams mirror the variations in healthcare access, and these altogether are translated in these two profiles based on KT activity^18^. Considering the ecological framework discussed above, access to health care, and hence to KT centers, can be considered a meso-level characteristic that influence adherence behaviors after receiving a kidney graft^12^. An improved quality of care provided by each KT center may, therefore, result in better clinical outcomes. Then, we believed the regional disparities offer a unique opportunity to study their influence on adherence to immunosuppressives and nonpharmacological treatment in KT recipients. Besides, the results could enable the design of individualized interventions to reduce the nonadherence rate in these settings.

This study aimed to estimate the prevalence and variability of nonadherence to immunosuppressives and nonpharmacological treatment across 20 KT centers and in the two health access-disparate regions in Brazil.

## METHODS

### Design, Sampling, and Setting

This study utilized data from the multicenter ADHERE BRAZIL study (*ClinicalTrials.gov* on 10/10/2013, NCT02066935), a cross-sectional study aimed at estimating the prevalence of nonadherence to immunosuppressives and nonpharmacological treatment among Brazilian transplant populations across different regions with different access to healthcare services. The study also included a comprehensive evaluation of factors associated with nonadherence in 20 KT centers in Brazil^21^.

The ADHERE BRAZIL study applied a multistage sampling strategy based on the transplantation activity of each center (low, moderate, and high) and the health access-disparate regions [Northeast/North/Mid-West (R1) and South/Southeast (R2)]^18,21^. The KT activity was based on a pre-existing classification for heart transplant^18^ and further submitted to a panel of Brazilian transplant nephrologists^21^. Transplant activity at the center level was defined as: low, less than 50 KTs/year; moderate, 50–150 KTs/year; and high, more than 150 KTs/year^21^. For the health access-disparate regions, R1 and R2, we considered the number of KTs performed per number of inhabitants during the last ten years^18^.

The 20 centers were chosen by convenience; the inclusion criteria involved obtaining consent for participation from their coordinators and the performance of at least 10 KTs per year over the five years preceding the start of the study (2010–2014)^21^. The selection of centers was also guided to keep a similar epidemiological profile when compared to the country. Patients scheduled for regular outpatient visit appointments were randomly selected based on the following inclusion criteria: age > 18 years, more than one post-transplant year, ability to understand the objectives of the study, and willingness to sign the informed consent form. The detailed description of the methodology (sample size, sample strata, theoretical framework, etc.) has been previously reported^12,21^ and adapted from the BRIGHT Study.^13^

### Variables and Measurements

The characteristics of the participating centers were assessed through structured interviews with their respective coordinators to obtain the following variables: number of beds available in the transplant center hospital [small/medium hospital (up to 150 beds); large hospital (151–500 beds); large specialized hospital (>500 beds)]; satisfaction with waiting room structure (yes *versus* no); patient follow-up by the same healthcare professional (yes *versus* no); multiprofessional team by Brazilian law [yes (doctor + nurse + nutritionist + psychologist + social worker) *versus* no]; continuous education of the KT team (yes *versus* no); clinical guidelines (yes *versus* no); clinical research (yes *versus* no); electronic medical records (yes *versus* no); and waiting room educational activities (yes *versus* no).^21^ The interview with patients included: satisfaction with the number of health professionals (yes *versus* no); difficulties in accessing the center by public transportation (yes *versus* no); time of medical consultation (15 *versus* 30 minutes); satisfaction with the schedule system of the transplant center (yes *versus* no); difficulties in scheduling appointments (yes *versus* no); adequacy of the frequency of consultation (yes *versus* no); adequacy of time of consultation(yes *versus* no)^21^.

The sociodemographic characteristics of patients were collected through interviews and medical records: age (years), sex (male or female), race (white *versus* non-white), marital status (steady partner *versus* not), education level [illiterate (0–4 years), elementary school (4–8 years), high school (> 8 to 11 years), college (> 11 years)], religion (Catholic, Protestant, Other), employment (active work *versus* no active work), and family income (up to 1 reference wage, > 1 to 3 wages, > 3 to 5 wages, more than 5 wages). Clinical data were collected by medical record review: pre-transplantation treatment time (months), pre-transplant treatment modality (hemodialysis, peritoneal dialysis, preemptive), donor type (living *versus* deceased donor), post-transplant time (years), latest creatinine levels, and episodes of acute rejection (yes *versus* no)^6,13,21^.

For the diagnosis of nonadherence to immunosuppressives, the implementation phase of medication adherence was evaluated using the Brazilian Portuguese validated version of the Basel Assessment of Adherence with Immunosuppressive Medication Scale (BAASIS)^22^. This self-report questionnaire is composed of four dimensions frequently associated with the failure of the implementation phase (taking and timing adherence, drug holidays, and dose reduction). Patients who reported any deviation in any of the items within the preceding four weeks were considered nonadherent^22^.

Nonadherence to nonpharmacological treatment components (physical activity, smoking cessation, alcohol consumption, and appointment keeping) was evaluated through the patient interview during routine office consultations^21^. A patient who performed less than 150 minutes of activity per week was considered nonadherent to the physical activity recommendations^23^. For smoking, we considered nonadherent those who consumed cigarettes during the data collection period^24^. nonadherence to alcohol ingestion was a daily alcohol consumption of one drink for women and two for men^25^. Regarding the frequency of consultations, we defined nonadherence as missing more than one of the last five scheduled appointments^21^.

### Data Collection

Data were collected from December 2015 to April 2017 during routine consultations in the transplantation service through the Research Electronic Data Capture system, a secure internet program that stores data and can be remotely powered by trained personnel^23^. All coordinators were trained to use the system for the data collection. Patients scheduled for consultation wererandomly selected by a computerized method, and those who were eligible would receive information about the ADHERE study to provide written consent^21^.

### Statistical Analysis

Categorical variables were described by frequency and percentage, while continuous variables by central tendency and dispersion measure, where appropriate.

Because the sample was designed to be self-adjusted^21^, the comparisons between regions (R1 and R2) in nonadherence to immunosuppressives and nonpharmacological treatment components (in terms of centers, demographics, and clinical data) were analyzed using the adjusted χ^2^ test or t-test. All variables are presented with their respective 95% confidence intervals (95%CI). The analyses were performed using STATA version 14 (StataCorp LP, College Station, TX, USA).

### Ethical Considerations

The study was approved by the Ethics Committee of the University Hospital of the Universidade Federal de Juiz de Fora (691,120) and registered nationally (CAAE 27972914.1.1001.5133). Participating centers also submitted the protocol for approval by their local ethics committee. All patients signed informed consent forms before data collection.

## RESULTS

### Sample Characteristics (Centers and Patients)

**Figure 1:**
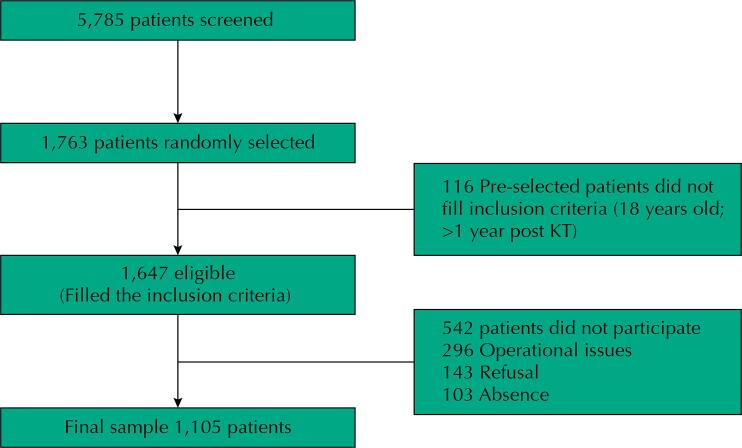
Flowchart of enrolment of KT patients. KT – kidney transplant.

At the 20 participating centers, 5,785 patients were screened. Of them, we randomly selected 1,763 patients; and from these, 1,647 met the inclusion criteria. We finally included 1,105 patients (participation rate, 67%). The remaining were excluded due to operational issues (n = 296), refusal for participation (n = 143), and nonattendance to the consultations (n = 103) (Figure 1).

Most centers of the ADHERE Brazil study (n = 17; 85%) were of low or moderate KT activity and located in large hospitals (53.6%). Dissatisfaction with the waiting room structure was frequently reported (42.7%). It was higher in R1 (71.9% *versus* 33.4%, p = 0.005), as was dissatisfaction with the number of health professionals (overall, 44.6%; 61% *versus* 39.6%, p = 0.01). The overall rate of consultation frequency adequacy was very high (86.4%), and this was higher in R1 (87.9 *versus* 86.1%, p = 0.02). The facility of immunosuppressives dispensing was considered too distant for most patients, mainly in R1 (66.9 *versus* 49.9%, p = 0.002). However, the vast majority underwent their laboratory examinations in public health system facilities (90.3%), more frequently in R2 than in R1 (92.6 *versus* 83.5%, respectively). Other variables were similar between regions (Table 1).

**Table 1:** Centers and health system characteristics data.

Variable	All regions (N = 1105) %(n)	95%CI	N/NE/MW (N = 267) %(n)	95%CI	South/Southwest (N = 838) %(n)	95%CI	p*
Center characteristics							
Number of beds of the transplant center hospital							
Small/medium hospital (till 150 beds)	7.8 (86)	1.92–26.6	7.5 (20)	5.7–53.1	7.9 (66)	1.4–32.4	0.26
Large hospital (151 to 500 beds)	53.6 (592)	26.9–78.2	81.0 (216)	31.8–97.4	44.9 (376)	26.9–78.2	
Special large hospital: > 500 beds	38.6 (427)	17.0–65.8	11.6 (31)	9.1–65.2	47.3 (396)	16–77.5	
Multiprofessional team, Brazilian law Doctor+ nurse + nutritionist + psychologist + social worker (yes)	80.1 (886)	50.8–94	92.5 (247)	46.8–99.4	76.2 (639)	41.0–93.6	0.59
Clinical guidelines (yes)	94.0 (1039)	74.9–98.8	100.0 (267)	100.0	92.1 (772)	67.5–98.5	0.48
Continuous education directed to KT team (yes)	97.7 (1080)	82.0–99.7	100.0 (267)	100.0	97.0 (813)	76.6–99.6	0.61
Electronic medical records (yes)	53.8 (595)	25.6–79.7	29.6 (79)	4.8–77.5	61.6 (516)	25.0–88.5	0.28
Waiting room educational activities (yes)	47.9 (529)	21.3–75.6	57.7 (154)	12.6 – 92.7	44.7 (375)	16.0–77.4	0.68
Not satisfied with waiting room structure* (yes)	42.7 (427)	32.7–53.4	71.9 (192)*	54.6 – 84.4	33.4 (280)*	25.3–42.7	0.005
Not satisfied with the number of health professionals* (yes)	44.6 (493)	44.9–64.9	61.0 (163)*	52.2 – 69.2	39.6 (330)*	27.4–53.1	0.01
Difficulties in accessing the center by public transportation (yes)	13.7 (152)	6.2–27.9	13.8 (37)	6.2 – 27.9	13.7 (115)	6.3–27.2	0.69
Average total time of medical consultation							
15 minutes	31.4 (348)	12.9–58.7	27.3 (73)	3.8–78.1	32.8 (257)	11.6–64.3	0.83
30 minutes	68.5 (757)	41.2–87.0	72.6 (194)	21.8–96.2	67.1 (563)	35.6–88.3	
Not satisfied with the schedule system of the transplant center (yes)	22.9 (253)	13.9–35.4	31.2 (83)	19.0–46.6	20.3 (170)	9.6–37.9	0.27
Difficulties in scheduling appointments (yes)	10.3 (114)	6.2–16.8	16.2 (43)	6.4–35.4	8.5 (71)	4.3–16.1	0.23
Adequacy of the frequency of consultation* (yes)	86.4 (955)	83.0–89.5	87.9 (234)*	81.3–92.4	86.1 (721)*	81.9–89.5	0.02
Adequacy of time of consultation (yes)	94.8 (1,046)	93.1–96.1	93.2 (248)	89.2–95.8	95.3 (798)	93.6–96.6	0.18
Health system							
Private insurance	23.4 (259)	19.3–28.0	18.7 (50)	12.3–27.3	24.9 (209)	20.4–30.0	0.18
Lab exams by public health system	90.3 (997)	83.6–94.5	83.5 (223)	75.8–89.1	92.6 (744)	84.5–96.2	0.06
Refill of immunosuppressives in another city	45.8 (506)	38.4–53.4	52.2 (139)	42.1–62.2	43.7 (367)	34.8–53.2	0.12
Immunosuppressives refill in a distant place*	54.0 (596)	46.3–61.5	66.9 (178)	62.9–70.6	49.9 (418)	40.5–59.3	0.002

Data are shown in mean + standard deviation or frequencies.

* We compare regions N/NE/MW (R1) *versus* South/Southwest (R2) by adjusted Chi-square test/95%CI or t test.

Fifty-eight percent of patients were 58.5% male (53.5% in R1 *versus* 60.1% in R2). Most of them self-declared as white (overall, 51.4%; 23.6 *versus* 60.2%). In R2, the patients were older than those in R1 (48.6 [SD = 12.2] *versus* 44.1 [SD = 13.1] years, respectively). Regarding education level, 39% had 4–8 years of schooling (32.2% in R1 *versus* 41.4% in R2). Ninety-three percent underwent hemodialysis before transplantation (97.0% in R1 *versus* 91.8% in R2), and 65.2% received a deceased donor graft in all regions. The mean overall creatinine level was 1.6 [SD = 0.83 mg/dL (1.4 [SD = 0.78 mg/dL] in R1 versus 1.6[SD = 0.84 mg/dL] in R2) (Table 2).

**Table 2:** Socio-demographic and clinical data of patients of the total sample and of health access-disparate regions

Variable	All regions (N = 1105) %(n)	95%CI	N/NE/MW (N = 267) %(n)	95%CI	South/Southwest (N = 838) %(n)	95%CI	p*
*Gender*							
Male	58.5 (647)	54.2–62.7	53.5 (143)	45.5–61.3	60.1 (504)	56.0–64.0	0.13
*Race*							
White*	51.4 (586)	41.1–61.5	23.6 (63)	16.6–32.3	60.2 (505)	46.2–72.8	0.001
*Age* (years)	47.5 ± 12.6	–	44.1 ± 13.1	–	48.6 ± 12.2	–	0.001
*Education level*							
Illiterate (0–4y)	7,8 (86)	4.9–12.0	6.3 (17)	3.2–12.0	8.2 (69)	4.7–13.9	0.15
Elementary school (4–8 years)	39.0 (431)	35.4–42.7	32.2 (86)	24.1–41.6	41.4 (345)	36.9–45.5	
High school (> 8 to 11 years)	38.3 (423)	33.9–42.8	43.0 (115)	35.9–50.4	36.7 (308)	31.1–42.7	
College (< 11 years)	14,9 (165)	12.3–17.9	18.3 (49)	15.2–22	13.8 (116)	10.8–17.5	
*Marital Status*							
Stable partner*	60.0 (662)	56.3–63.5	54.3 (144)	48.0–60.5	61.8 (518)	57.8–65.6	0.04
Not employed*	76.7 (848)	73.3–79.8	81.6 (218)	76.9–85.5	75.1 (630)	71.3–78.6	0.03
*Family income*							
Until 1 reference wage	25.4 (281)	19.2–32.8	33.0 (88)	23.1 – 44.4	23.0 (193)	16.6–31.0	0.16
> 1 to 3 wages	52.7 (582)	46.4–58.8	50.9 (134)	43.3–57	53.5 (448)	45.8–61.0	
> 3 to 5 wages	14.2 (157)	11.4–17.5	9.7 (26)	5.2–17.4	15.6 (131)	13.0–18.6	
Up to 5 wages	7.6 (84)	4.9–11.4	7.1 (19)	4.8–10.3	7.7 (65)	4.5–13.0	
*Religion*							
Catholic	63.6 (703)	60.6–66.5	68.5 (183)	65.5–71.3	62.0 (520)	58.4–65.5	0.45
Protestant	26.0 (287)	23.3–28.7	24.3 (65)	19.2–30.2	26.4 (222)	23.3–29.8	
Other	10.5 (116)	1.0–3.6	7.2 (19)	1.0–11.1	11.4 (96)	1.2–3.8	
Pre-KT treatment*	93.0 (1028)	91.3–94.4	97.0 (259)	95.1–98.1	91.8 (769)	89.7–93.3	0.05
Hemodialysis	3.7 (40)	2.4–5.2	1.9 (5)	0.7–5.1	4.2 (35)	2.9–5.9	
Peritoneal dialysis	3.3 (37)	2.2–5.1	1.1 (3)	0.3–4.3	4.0 (34)	2.5–6.4	
Preemptive							
*Pre-transplantation treatment time (months)*	40.4 ± 40.4	–	42.7 ± 39.8	–	39.6 ± 40.5	–	0.13
*Post-transplant time (years)*	6.2 ± 4.8	–	6.0 ± 4.9	–	6.2 ± 4.7	–	0.77
*Type of donor*							
Deceased	65.2 (721)	55.8–73.5	72.2 (193)	54.2–85.1	63.0 (528)	52.9–72.1	0.32
*Acute rejection*							
Yes	22.8 (2)	17.4–29.3	19.4 (52)	15.1–24.6	23.9 (198)	17.1–32.2	0.29
Last creatinine*	1.6 ± 0.83	–	1.4 ± 0.78	–	1.6 ± 0.84	–	0.001

Data are shown in mean + standard deviation or frequencies.

* We compare regions N/NE/MW (R1) *versus* South/Southwest (R2) by adjusted Chi-square test/95%CI or t test.

### Prevalence of nonadherence to immunosuppressives and health behaviors

**Figure 2:**
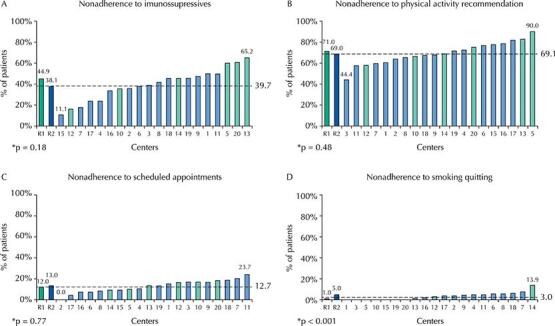
Nonadherence to immunosuppressives (2A) and nonpharmacological treatment (2B: physical activity recommendations, 2C: scheduled appointments and, 2D: smoking cessation) in the total sample (dotted line), at each center (numbers in horizontal axis) and in health access-disparate regions. R1 = Northeast/North/Mid-West (green) and R2 = South/Southwest (orange). We compared the R1 *versus* R2 regions by adjusted Chi-square test.

**Figure 3:**
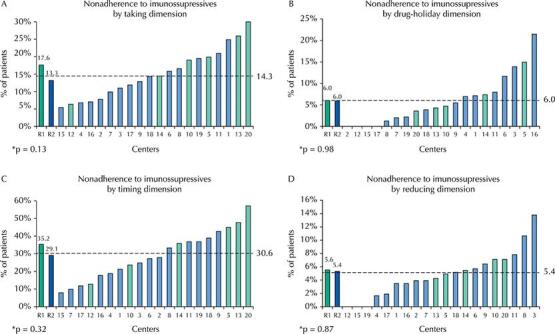
Implementation nonadherence to immunosuppressives by BAASIS© scale dimensions [taking (2A), timing (2B), drug holidays (2C) and dose reduction (2D)] in total sample (dotted line), at each center (numbers in horizontal axis) and in health access-disparate regions. R1 = Northeast/North/Mid-West (green) and R2 = South/Southwest (orange). We compared the R1 *versus* R2 regions by adjusted Chi-square test.

The overall prevalence of nonadherence to immunosuppressives was 39.7% (range, 11.0–65.2%) among KT centers (Figure 2). When evaluating nonadherence to immunosuppressives by the four separate dimensions of the BAASIS^©^ (taking and timing, drug holidays, and dose reduction), the highest prevalence in the sample was in the deviations in timing (30.6%), followed by nonadherence to immunosuppressives intake (14.3%), drug holiday (6.0%), and dose reduction (5.4%) (Figure 3).

Regarding nonpharmacological treatment components, the highest prevalence of nonadherence was to physical activity recommendations (less than 150 min/week), accounting for 69.1% (range, 44.5–90%). Nonattendance of at least one of the last five scheduled appointments was 12.7% (0–23.7%). Only 3.9% (0–13.9%) were current smokers, and all participants denied being heavy drinkers (Figure 2).

Comparing regions and the prevalence of nonadherence to nonpharmacological treatment, only nonadherence to smoking cessation was more frequent in the R1 region (5% *versus* 1%; p < 0.001) (Figure 2).

## DISCUSSION

This was the first multicenter study of KT patients to evaluate adherence to different health behaviors in Brazil. It is also the first study elucidating a panel of these risky behaviors in a variety of KT centers across the two health access-disparate regions of Brazil. We deemed the large sample size as one of the strengths of our study, alongside the sample design that aimed to approximately represent the Brazilian KT population, and the use of a theoretical framework, i.e., the ecological model^12^, to select potential multilevel correlates, which is uncommon in existing adherence studies.

The studied sample has demographic characteristics that reflect the KT population worldwide – age, male sex, deceased-donor type, and hemodialysis as previous treatment. We found that the schooling level and family income represented a specific epidemiological frame, particularly of a low socioeconomic level population. Furthermore, for the first time, some aspects of the clinical practice of KT centers have been analyzed and compared based on two healthcare access-disparate regions in Brazil. The higher dissatisfaction with the waiting room structure and with the number of health professionals reported by patients from R1, which supposedly had worse access, reinforced the hypothesis of limited health care availability, such as the greater distance to immunosuppressives dispensing facilities.

The highest frequency of nonadherence after KT in the entire sample and the studied regions was to physical activity. In one center, 90% of patients were physically inactive. Despite being understudied, the nonadherence to post-KT physical activity was estimated at 35–84%^8,26,27^, similar to what we found in our study. Physical activity helps prevent cardiovascular diseases, which is the major cause of post-transplant death and improves the quality of life^28^. Although physical activity promotion programs have improved some aspects of physical performance, the evidence for their effects on graft outcomes is lacking. Therefore, physical activity programs are a promising area of research^28^.

The second highest prevalence of nonadherence was to immunosuppressives. We found a total prevalence of 39.7%, similar to the previously reported 20–60%^1,4,5^, using self-report instruments for diagnosis. Interestingly, we found some effect, with the additional 7 points in the prevalence of R1 and a p-value of 0.18, nonadherence to immunosuppressives was similar in the two health access-disparate regions. There are currently no data on access to healthcare services for KT in Brazil. The universal access to health services provided by our public health system is challenged in some low socioeconomic level areas, which is reflected by the smaller number of consultations and health professionals^29^. The treatment of chronic kidney disease in its more advanced phase, — dialysis and transplantation— is classified as highly complex, has differentiated reimbursement, and is often provided by a well-organized center/hospital. Thus, after KT, the patient has the opportunity to be followed in such a structured service, surpassing the other failures of the health system. However, we found no association between R1 and R2 in terms of the four BAASIS dimensions (immunosuppressives taking and timing, drug holidays, and dose reduction). Further studies, applying different methodologies to categorize health access, such as the five geographic regions or the human development index, could better explore this finding.

We found a high prevalence of patients not keeping their appointments for KT services (12.7%). This figure varied widely among centers but not between the studied regions. This prevalence is one of the highest among the few available studies^5,11^. Strategies for decreasing nonattendance are feasible and could prevent this behavior, which is ultimately associated with graft loss^11^.

Nonadherence to smoking cessation was similar to that described by other KT studies, but this behavior was only one more frequent in R2. The highest nonadherence rate among services was 13.9%. In this case, the higher prevalence of smokers in the R2 region can be attributed to the relatively better economic status of patients, favoring access to cigarettes^30^. Smoking is associated with an increased risk of cardiovascular death and a 50% greater risk of graft loss when compared to ex-smokers. These findings suggest the need for greater emphasis on smoking cessation, even in the pre-transplantation period^9^.

Alcohol dependence in the pre-transplantation phase is associated with a 38% increased risk of graft loss due to death and a 56% increased risk of transplant-related death^10^. None of the studied KT patients reported the use of alcoholic beverages. The methodological rigor used to define this NAd behavior may have influenced these results^25^.

The prevalence of nonadherence to treatment dimensions reported in the KT population supports that services include specific interventions to encourage adherence to immunosuppressives and to relevant nonpharmacological treatment components such as physical activity and smoking cessation. KT centers should also emphasize the importance of attendance to consultations for controlling the current health condition^7,9,10^. Evidence of interventions to reduce nonadherence is limited, but studies suggest that efficient measures should be multidimensional and involve a multiprofessional team (doctors, nurses, pharmaceutics, psychologists, nutritionists, social assistants). Strategies for assessing specific individual barriers and developing measures that could be integrated into daily life have also been proposed. These personalized care interventions directed to improve self-care management, reinforce self-monitoring and self-efficacy and have the potential to improve adherence to all aspects of treatment included in our study^15,16^.

The first limitation of this study is that the cross-sectional design did not allow for causal inferences. Second, the 20 participating centers were selected by convenience to ensure economic viability and minimize data loss. In the attempts to avoid selection biases, patients were selected randomly for inclusion, but we only screened those with scheduled appointments during the data collection period. Finally, we diagnosed nonadherence using a self-report method, which may have led to underestimation in results^22^.

## CONCLUSION

In this large-sample study, which approached to be representative of the Brazilian KT population, there was an overall high prevalence of nonadherence to immunosuppressives and to nonpharmacological treatment (physical activity, smoking cessation, and keeping appointments). Differences were found among clinical practices in the two health access-disparate regions, while only nonadherence to smoking cessation occurred more frequently in the R2 region. We propose that the quality of healthcare during follow-up surpassed the limitations in healthcare facilities, which could have potentially influenced the prevalence of nonadherence among KT patients. Nonetheless, transplant service professionals should consider seeking interventions to reduce nonadherence since they may negatively impact post-transplant outcomes.
